# The systemic oral health connection: Biofilms

**DOI:** 10.1097/MD.0000000000030517

**Published:** 2022-11-18

**Authors:** Gregori M. Kurtzman, Robert A. Horowitz, Richard Johnson, Ryan A. Prestiano, Benjamin I. Klein

**Affiliations:** a Private dental practice, Silver Spring, Maryland, USA; b Private periodontal practice Scarsdale, New York, USA; c Adjunct Clinical Assistant Professor, Department of Periodontology and Implant Dentistry, New York University College of Dentistry; d Private medical practice, New York, New York; e Clinical Research Associate Periotech, Briarcliff Manor, New York; f Pre-medical student, Middlebury College, Middlebury, Vermont.

**Keywords:** Alzheimer’s syndrome, cardiovascular disease, chronic kidney disease, colon cancer, diabetes, erectile dysfunction, Oral biofilm, pancreatic cancer, prostate disease, pulmonary disease

## Abstract

Frequently, periodontal health and it’s associated oral biofilm has not been addressed in those patients who have systemic health issues, especially those who are not responding to medical treatment via their physician. Oral biofilm may be present in the periodontal sulcus in the absence of clinical disease of periodontal disease (bleeding on probing, gingival inflammation) and periodontal reaction is dependent on the patient’s immune response to the associated bacterial and their byproducts. Increasing evidence has been emerging the past decade connecting oral biofilm with systemic conditions, either initiating them or complicating those medical conditions. The patient’s health needs to be thought of as a whole-body system with connections that may originate in the oral cavity and have distant affects throughout the body. To maximize total health, a coordination in healthcare needs to be a symbiosis between the physician and dentist to eliminate the oral biofilm and aid in prevention of systemic disease or minimize those effects to improve the patient’s overall health and quality of life. Various areas of systemic health have been associated with the bacteria and their byproducts in the oral biofilm. Those include cardiovascular disease, chronic kidney disease, diabetes, pulmonary disease, prostate cancer, colon cancer, pancreatic cancer, pre-term pregnancy, erectile dysfunction Alzheimer’s disease and Rheumatoid arthritis. This article will discuss oral biofilm, its affects systemically and review the medical conditions associated with the oral systemic connection with an extensive review of the literature.

## 1. Introduction

**Figure 1.**
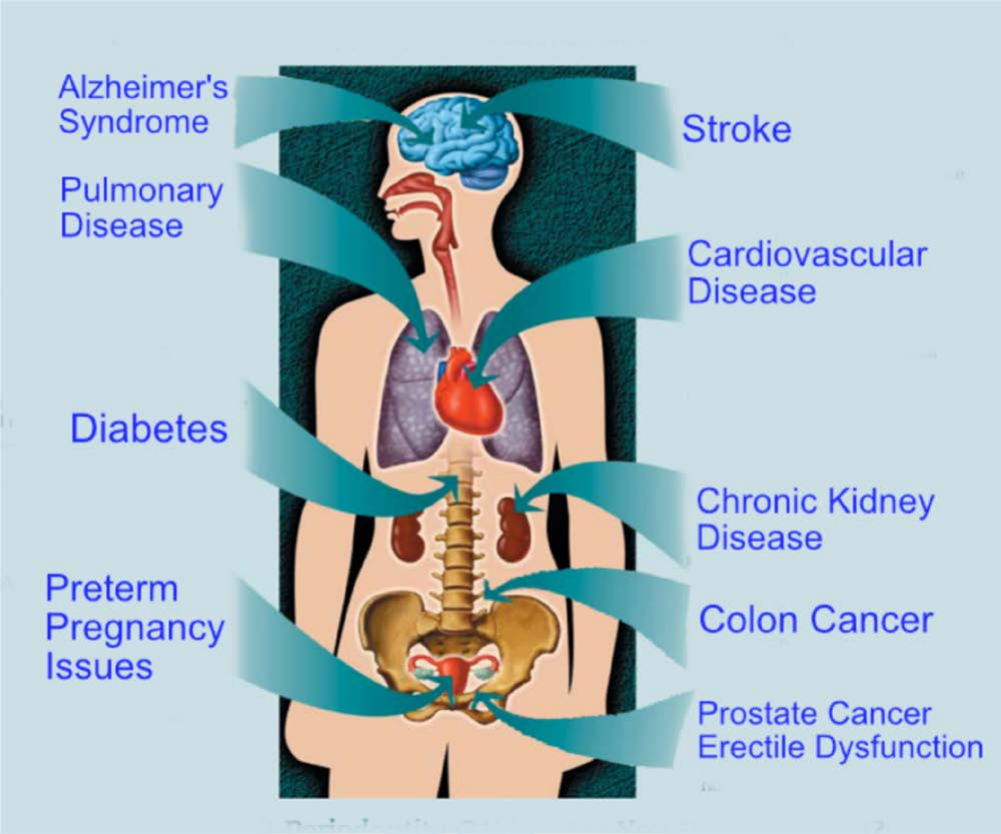
Systemic issues that have been connected to oral biofilm.

Family physicians are in a unique position to access and treat the patient holistically. As they are managing the patient’s systemic health, they may be the first healthcare practitioner to identify the presence of systemic issues or see management issues with those systemic health issues. Increasing evidence has been emerging the past decade connecting oral biofilm with systemic conditions, either initiating them or complicating those medical conditions. (Figure 1) The patient’s health needs to be thought of as a whole-body system with connections that can originate in the oral cavity and have distant affects throughout the body. To maximize total health, a coordination in healthcare needs to be a symbiosis between the physician and dentist to eliminate the oral biofilm and aid in prevention of systemic disease or minimize those effects to improve the patient’s overall health and quality of life. This article will discuss oral biofilm, its affects systemically and review the medical conditions associated with the oral systemic connection.

## 2. What is an oral biofilm?

Dental plaque, what has been re-termed “oral biofilm”, has long been connected with periodontal problems and to a lesser extent caries related to the bacteria contained within it. That oral bacterium, was long ignored for any affects outside the oral cavity. Yet, research has been accumulating directly connecting a link between oral health and systemic disease, with 200 possible connections between systemic diseases and oral health reported by The American Dental Association.^[1]^ Accumulating evidence has linked periodontal disease and chronic oral inflammation to multiple health conditions, including cardiovascular and renal issues, diabetes, osteoporosis, pulmonary disorders, Alzheimer’s, and other systemic conditions. With that research in mind, oral biofilm has been recognized as a more complex environment than previously understood.^[2,3]^

Oral biofilm is a community of microorganisms found on the tooth surface or within the sulcus (periodontal pocket) which are embedded in a matrix of polymers of host and bacterial origin. Typically, more than 700 different species of bacteria naturally reside in the mouth, with most considered innocuous, but some of these microorganisms have been identified as pathogenic. As bacteria increase in number, they quickly create an intricate network of protective layers (ie, matrix) and channels that develops into a biofilm and are major cause of periodontal disease. Those bacteria in the biofilm are less susceptible to antimicrobial agents, either locally applied or systemically administered. It has been long demonstrated that these microbial communities can display enhanced pathogenicity (pathogenic synergism).^[4,5]^ Additionally, the structure of the biofilm might restrict penetration of antimicrobial agents, while bacteria growing on a surface (planktonic) are susceptible to antimicrobial agents.^[6]^ The aggregation of bacteria works together as a community, producing specific proteins and enzymes by way of quorum sensing, utilizing oral fluids as the vector for transmission.^[7]^ Bacteria in these oral environments have evolved as part of multispecies biofilms and can require interaction with other bacterial species to grow, forming complex bio-environments.^[8]^

The oral biofilm bacteria have the ability to regulate numerous processes via quorum sensing, which is a cell-to-cell communication mechanism that synchronizes gene expression in the biofilm in reaction to the density of the cell population.^[9]^ This includes secreting specific enzymes to activate or deactivate the genes of other bacteria. These bacterial byproducts provoke an immune response from the host, which recruits white blood cells (WBC) to the site to kill the invading bacteria, which results in localized inflammation in the surrounding gingiva. Using quorum sensing, the bacteria have the ability to confuse the defending WBC chemotactically by releasing chemicals into the local environment, rendering the immune response ineffective. As WBCs have a 3-day life cycle, if they do not engulf a bacterium and destroy it within that time frame, they lyse and die.^[10]^ So components within the WBC that were intended to kill the bacteria are now available to damage the very tissue they were meant to protect contributing to periodontal bone loss and a deepening of the pockets and inflammation.^[11]^ The composition of the bacterial community in the biofilm is diverse, with variations in the many species being detected which can be different from site to site in the same patient. Once formed, the species composition at a site is characterized by a degree of stability among the component species due to a balance between synergism and antagonism.^[12]^ The oral biofilm is most likely to be seen in its mature state in periodontal pockets as these areas provide protection from removal with homecare by the patient.^[13,14]^ As the biofilm matures, the microbial composition changes from one that is primarily gram-positive and streptococcus-rich to a structure filled with gram-negative anaerobes.^[15]^

Initially the biome of the biofilm consists of mostly gram-positive cocci bacteria (Streptococcus mutans, Streptococcus mitis, Streptococcus sanguis, Streptococcus oralis, Rothia dentocariosa, and Staphylococcus epidermidis), followed by some gram-positive rods and fillaments (Actinomyces viscosus, Actinomyces israelis. Actinomyces gerencseriae and Corynebacterium species) and a very small number of gram-negative cocci. Veillonella parvula and Neisseria species making up some of the gram-negative cocci, are aerobes or facultative aerobes. This early biofilm is able to withstand frequent mechanisms of the oral bacterial removal such as chewing, swallowing and salivary fluid flow. These early colonizers are also able to survive in the high oxygen concentrations present in the oral cavity. This initial biofilm, always present orally, forms immediately after cleaning. Co-adhesion of later bacterial colonizers to the initial biofilm continues to involve specific interactions between bacterial receptors, this builds up the biofilm creating a more complex and diverse environment. Those diverse bacterial species create synergistic and antagonistic biochemical interactions amongst the colony’s inhabitants. This can contribute metabolically with other bacteria that are located physically close to them. When obligate aerobes and anaerobes are involved in co-adhesion, these interactions can ensure the anaerobic bacteria’s survival in the oxygen-rich oral cavity. The bacteria continue to divide until a three-dimensional mixed-culture biofilm forms that is specially and functionally organized. Polymer production causes development of an extracellular matrix. This matrix is a key structural aspect of the biofilm offering the inhabitants protection from external factors. As the biofilm thickens and becomes more mature, those anaerobic bacteria are able live deeper within the biofilm, further protecting them from the oxygen-rich environment within the oral cavity.

Yet, oral biofilm does not need to present with gingival bleeding to create issues systemically and identification of the presence intraorally is directed by the dentist to identify its presence whether periodontal disease is present or not. The presence of periodontal disease varies by the patient’s immunological response to the bacteria in the biofilm. Some patients will present with typical signs of periodontal disease such as bleeding on probing or brushing and gingival inflammation. Whereas others will not present with these typical signs.

## 3. The systemic connection

Harmful strains of bacteria in the oral biofilm can enter the bloodstream during the inflammatory response and can travel to other areas of the body, exerting a distant systemic effect that has been linked to numerous diseases. Increasing evidence indicates patients with periodontal disease, have a much higher risk of developing cardiovascular and other systemic issues than those individuals who take preventive measures to eliminate and control the biofilm in their mouths.^[1]^

## 4. Cardiovascular disease (CVD)

CVD, an umbrella term for heart and blood vessel conditions, such as atherosclerosis, coronary heart disease, stroke and myocardial infarction is the result of a complex set of genetic and environmental factors.^[16,17]^ There is increasing evidence linking chronic infection and inflammation to CVD with the biofilm as a predisposing factor.^[18,19]^ The connection between oral bacteria and cardiac disease is not a recent development in the literature. Oral bacteria, specifically *Streptococcus mutans* (cariogenic) and *Porphyromonas gingivalis* (periodontitis) induce platelet aggregation, leading to thrombus formation.^[20]^ One or more periodontal pathogens as reported in the literature are found in 42% of atheromas in patients with severe periodontal disease.^[21]^ It has been reported that *P gingivalis* actively can adhere to and invade fetal bovine heart endothelial cells and aortic endothelial cells.^[22]^ Additionally, a 14-year study found periodontal disease patients had a 25% higher risk to develop CVD than their orally healthy counterparts.^[23]^ Men younger than 50 years with periodontal disease demonstrate 72% more risk to develop CVD. Additionally, periodontal disease increased risk for both fatal and non-fatal strokes two-fold.^[24]^ Despite the strong evidence of an association between periodontal disease and CVD, it is unknown if it is a direct or causal relationship.

Periodontal disease releases bacteria that can enter the circulation, invading the heart and vascular tissue, causing harmful effects. People with higher levels of bacteria in their mouths tend to have thicker carotid arteries, an indicator of CVD.^[25]^ Bacteria near diseased gingiva appears to induce clumping of blood platelets, which can then cause the clotting and blockages that can lead to heart attacks or strokes. The body’s response to periodontal infection includes production of inflammatory mediators, which travel through the circulatory system and will cause harmful effects on the heart and blood vessels. Inflammatory mediators such as lipoprotein and triglycerides are significantly higher in patients with periodontitis than in control groups.^[26]^ Increased levels of C-reactive protein, a biomarker for inflammation is associated with periodontitis shows increased clotting which is associated with an elevated risk of heart disease.^[27]^ Periodontal disease’s emergences as a potential risk factor for CVD is leading to a convergence in oral and medical care. Proper management of oral health should very well be key to prevention of cardiac disease or worsening of existing heart conditions.^[28,29]^

## 5. Chronic kidney diseases (CKD)

CVD and CKD share many risk factors, with periodontal inflammation linked to the development of kidney disease.^[30]^ Pathogens found in oral biofilms have been shown to have the ability to adhere to and invade coronary endothelial cells, leading to atheroma formation and impaired vasculature relaxation, with similar effects noted within the vasculature of the kidney.^[31]^ CKD most common causes are diabetes mellitus, hypertension, and glomerulonephritis, which together cause approximately 75% of all adult cases.^[32]^ CKD patients are characterized by a few well-established risk factors of periodontal disease, including poor oral hygiene and diabetes.^[33]^ A strong correlation appears to exist between patients on dialysis and the high number of patients suffering from gingivitis (46%) and severe periodontitis (35%).^[34]^ The correlation appears to be bi-directional, as patients with CKD have higher prevalence of periodontal disease.

## 6. Diabetes

Those patients with diabetes have twice the risk for periodontal disease of those without the metabolic disorder.^[35]^ In addition, periodontal disease is more prevalent, progresses more rapidly, and is often more severe in patients with type I or type II diabetes.^[36,37]^ Periodontal disease has been classified as the sixth most common complication of diabetes and is a strong, well-established risk factor for severe periodontal disease. Patients with periodontal infections have worse glycemic control over time and thus have greater difficulty managing their diabetes. Treatment of periodontitis appears to improve glycemic control.^[38]^ Thus, control of the periodontal infection and associated biofilm should be part of the standard treatment for the diabetic patient. Those patients who are having difficulty managing their diabetes via medication or even those who are using a dietary approach, should be referred for a dental periodontal evaluation and management of the oral biofilm. It has been found that oral biofilm management improved diabetes and aided patients in their management of this disease.

## 7. Pulmonary disease

Periodontal biofilm is a reservoir of bacteria and a source of lower airway infections, especially in older patients or those who are debilitated inoculating the respiratory tract when aspirated. Severity of the disease is correlated with the pathogenicity of the bacteria in the biofilm. Periodontal pathogens and cariogenic bacteria increase risk factors for aspiration pneumonia.

The highest risk patients for respiratory infection (bronchitis and pneumonia) are medically compromised patients with or without respiratory disease who are unable to perform adequate oral homecare. Evaluation of 328 articles published over an 11-year period reported linking oral hygiene to oral health care–associated pneumonia or respiratory tract infection in elderly patients. Evidence indicates homecare oral hygiene practices reduce the progression or occurrence of respiratory diseases in high-risk elderly people in nursing homes or hospitals. Those oral hygiene practices should prevent the death of about 1 in 10 elderly residents of nursing homes from health care–associated pneumonia.^[39]^ Proper oral homecare is critical in preventing these oral infections by minimizing the potential of aspirating biofilm into the pulmonary system. One author reported, “Oral hygiene intervention significantly reduced occurrence of pneumonia in institutionalized subjects.”^[40]^ Frequent tooth brushing and preoperative use of 0.12% chlorhexidine mouthrinse or gel reduced nosocomial respiratory tract infections.^[41]^ Additional evidence that the use of chlorine dioxide oral rinses, available OTC in most drug stores or supermarket with other oral care products reduces oral biofilm intraorally and rinsing removable prosthetics (dentures) in this eliminates biofilm decreasing aspiration potential.^[42,43]^ Therefore, placing all elderly patients on chlorhexidine or chlorine dioxide daily rinses as a preventive measure should be recommended to aid in preventing potential aspiration pneumonia. This should be a more predictable approach in elderly patients who lack manual dexterity to perform oral homecare with a toothbrush.

Those patients who are elderly, tend to also be denture wearers (higher incidence compared to younger populations) and can be susceptible to aspiration of biofilm growing on the denture. As this patient population has decreased immune systems and an increase in systemic issues, they are particularly susceptible to pulmonary infections which can be induced via the bacteria within the biofilm attached to the denture. This becomes more problematic in the nursing home setting or for those patients unable to care for their own dentures due to either physical or mental limitations. This can be performed daily by nursing staff or homecare aids to help prevent potential aspiration of biofilm in this compromised population.

## 8. Prostate disease

Prostate-specific antigen (PSA) has been reported to be secreted at much higher levels in men with periodontal disease.^[44]^ Inflammation of the prostate or when infection is present or affected by cancer demonstrates elevated PSA levels. Research has demonstrated that men with indicators of periodontal disease and prostatitis have higher levels of PSA than men who do not have periodontal disease.^[45,46]^

## 9. Colon cancer

The bacteria *Fusobacterium nucleatum*, found in the mouth and in periodontal biofilm, has a role in periodontal disease colonizing the gut and attaching to cells in the colon, triggering a sequence that can lead to colon cancer. It has been reported that patients with periodontal disease have much higher levels of *F. nucleatum* then those with normal periodontal status.^[47,48]^ Although a possible association was found between oral infection and colon cancer, a cause-and-effect relationship has not been found. Published studies show how *F. nucleatum* can speed the accumulation of cancer cells.^[49,50]^ Minimizing *F. nucleatum* by controlling and management of the oral biofilm can lower the risk for those who are at increased risk of developing colorectal cancer.

## 10. Pancreatic cancer

Pancreatic cancer risk factors include cigarette smoking and chronic pancreatitis, but the role of inflammation from periodontal disease can promote this cancer.^[51]^ Harvard School of Public Health and Dana-Farber Cancer Institute researchers found that periodontal disease can be associated with an increased risk of cancer of the pancreas. Additionally, research shows men with periodontal disease had a 63% higher risk of developing pancreatic cancer compared to those reporting no periodontal disease.^[52]^

## 11. Pre-term pregnancy

Evidence links an association between the presence of periodontitis and preterm delivery and low birth weight infants. Biofilm inflammatory molecules can enter the circulatory system and cross the placenta to reach the fetal membranes and cause preterm delivery, with oral bacteria identified in fetal membranes. The inflammation of the periodontal tissues due to the formation of the biofilm increases dramatically in size and severity during the course of a normal pregnancy.^[53]^ Mechanisms have been suggested to explain how periodontal disease can influence preterm, low-birth-weight babies. Lipopolysaccharides from cell walls of periodontal pathogens can trigger production of prostaglandins, periodontal infection leads to release of these prostaglandins into the circulatory system. Translocation of these periodontal bacteria to the fetus occurs via the placenta which stimulates the release of prostaglandins.^[54]^ These prostaglandins stimulate oxcytocin production which can initiate pre-term labor and result in lower birth weight babies.

## 12. Erectile dysfunction (ED)

ED a multifactorial condition, has been linked to organic (hormonal, vascular, drug induced), psychological causes, or a combination of both.^[55,56]^ However, the most common recognized cause of ED is vascular disease. Increased risk of endothelial dysfunction can be associated with high levels of inflammatory mediators such as interleukin (IL)-6, IL-8, tumour necrosis factor-alpha (TNF-α), and IL-1.^[57,58]^ TNF-α has been reported to play a key role in the induction of endothelial dysfunction.^[59]^ Multiple studies have shown a reduction in TNF-α levels after successful periodontal treatment, and evident that significant higher plasma levels of TNF-α were associated with moderate to severe ED owing to their known effects on the vasculature.^[60,61]^ Management of the oral biofilm and periodontal health can therefore have positive implications in management of ED.

## 13. Alzheimer’s syndrome

Bacterial agents including periodontal pathogens have recently been reported to be associated as important actors in Alzheimer’s disease pathology. Those microbiotas include Porphyromonas gingivalis (P. gingivalis), Prevotella melaninogenica (P. melaninogenica) and Campylobacter rectus (C. rectus), Prevotella nigrescens, Fusobacterium nucleatum, Streptococcus intermedius, Capnocylophaga Ochracea, and P. melaninogenica. There is increasing evidence of an association between periodontal pathogens and Alzheimer’s disease, especially in the older population.^[62]^ Those periodontal pathogens and the subsequent chronic inflammatory responses have significant implications on the development of Alzheimer’s disease. Data demonstrates periodontitis is associated with an increase in cognitive decline in Alzheimer’s Disease, which can be mediated through effects on systemic inflammation.^[63]^ The exact mechanism of periodontal pathology involved in the pathogenicity of Alzheimer’s disease is not currently known. Those bacteria can alter the host immune response in Alzheimer’s disease.^[64]^ Management of periodontal disease and its inflammatory mediators should slow progression of cognitive decline and extend the patient’s quality of life.

## 14. Rheumatoid arthritis (RA)

Rheumatoid arthritis affects 1-3% of the population, with evidence of a genetic predisposition to the disease. Rheumatoid arthritis is characterized by progressive and irreversible synovial-lined joint damage leading to loss of joint space, bone and joint function, leading to a structural deformity.^[65,66]^ Disease onset is either acute or subacute in 25% of those affected patients, with early onset characterized by symmetric polyarthritis involving the joints of the hands and feet with no radiologic changes. Periodontitis being a destructive inflammatory disease of the dental supporting tissues, severe periodontitis affects 5-15% of the adult population with lesser levels affecting larger portions of the population depending on their age. It has been reported that 47.2% of adults aged 30 years and older have some form of periodontal disease, which increases with age, with 70.1% of adults 65 years and older having periodontal disease. This is more common in men than women (56.4% vs 38.4%), and current smokers (64.2%).^[67,68]^ As a correlation has been reported between Rheumatoid arthritis and periodontal disease, coordinated treatment should be considered in those patients who have been diagnosed with Rheumatoid arthritis or are seeing the initiation of those joint changes. One study reported a prevalence of 45% of severe periodontitis in Rheumatoid arthritis compared to those patients with Osteoarthritis (33%).^[69]^ They further reported that severity of periodontitis was significantly higher among the patients with established Rheumatoid arthritis compared to those patients with Osteoarthritis.

Interactions of *P. gingivalis*, a prominent oral pathobionts, with host cells, which includes epithelial cells, phagocytes, and stem cells present in dental tissues have been reported.^[70]^ A previously unknown interaction of *P. gingivalis* bacteria with human stem cells that has an impact on human immune thereby showing a link between periodontitis and rheumatoid arthritis. Those patients with Rheumatoid arthritis are more likely to present with periodontal disease, concurrent poorer oral hygiene manifesting as an increased accumulation of oral biofilm and its associated bacteria and decreased salivary flow rates.^[71]^ So, it becomes a vicious cycle, increased oral biofilm leads to increased periodontal disease which either precipitates rheumatoid arthritis or exacerbates it causing a worsening of the periodontal disease that further enhances the rheumatoid arthritis issues. Those patients would benefit from improved periodontal care to eliminate and control the oral biofilm and thus its associated periodontal disease improving management and affects of rheumatoid arthritis.

## 15. The oral gut connection

The oral cavity and gut have the two largest microbial habitats that are interconnected, playing a major role in microbiome-associated diseases.^[72]^ The oral-gut connection may be a route linking periodontal and systemic diseases, with strong correlations reported between oral and fecal bacterial species. Evidence is emerging suggesting that periodontitis-associated pathogens can translocate to distant sites to elicit severe local and systemic pathologies, which necessitates research into future therapies. Fecal microbiota transplantation, probiotics, prebiotics, and synbiotics represent current modes of treatment to reverse microbial dysbiosis through the introduction of health-related bacterial species and substrates.^[73]^

Oral pathogens can disseminate to distant body organs via the local, oral blood circulation, or pass through the gastrointestinal tract, entering the systemic circulation. Once those oral pathogens reach an organ, they modify the immune response and stimulate the release of inflammatory mediators, resulting in systemic disease.^[74,75]^ Dissemination of those oral microbes to the gut may exacerbate various gastrointestinal diseases, including irritable bowel syndrome, inflammatory bowel disease, and colorectal cancer. However, the precise role that oral microbes play in the extraoral organs, including the gut, remains elusive.^[76]^ This correlates with decreased diversity of oral and gut microbiota playing an important role in the etiopathogenesis of rheumatoid arthritis and osteoarthritis.^[77]^

Although systemic probiotics combined with subgingival instrumentation did not provide short-term additional clinical or microbiological benefits in the treatment of periodontitis, response to treatment appeared to correlate with distinct oral-gut microbial profiles.^[78]^ Further research is needed to confirm the oral-gut connection and its affect on systemic health related to the bacteria and their byproducts found in these interconnected environments.

## 16. How can oral biofilms be managed?

Biofilm management involves treatment by the dentist to identify periodontal disease, manage that disease process and return the periodontal tissue to a more normal healthy state. But also involves improved homecare by the patient to keep biofilm levels down, prevent periodontal disease resurgence and the systemic effects that have been associated with it.

Mechanical debridement of the pocket is unable to remove all of the biofilm as toothbrushes are poorly effective more then 4mm subgingival under the best efforts of the patient. But, re-growth of the biofilm occurs within three hours resulting in a four-fold (400%) increase in biofilm mass.^[79]^ Homecare which is compromised no matter how diligent the patient tries to be as the toothbrush bristles are unable to extend more then 3-4mm into the pocket and is unable to mechanically contact the biofilm located at deeper depths. A similar problem presents with oral irrigators (ie. Waterpik and similar devices) not allowing irrigation to the bottom of the pockets and most patients are not diligent in its daily use. The sulcular environment is difficult for most patients to reach with brushing and flossing making it impossible to control oral biofilms by mechanical means alone as the bacteria grow and replicate so rapidly. Post-cleaning biofilm redevelopment is more rapid and complex, exceeding pre-cleaning levels within two days.^[80,81]^

Chlorhexidine has been reported to have an affect on young biofilms but the bacteria in mature biofilms and nutrient-limited biofilms have been shown to be more resistant to its affects.^[82,83]^ As previously discussed, chlorine dioxide has been demonstrated as an effective oral rinse that functionally debrides the biofilm slime matrix and bacterial cell walls, essentially peeling the biofilm back layer by layer. Chlorine dioxide has not been reported to have any negative effects either dentally, or systemically and is safe for daily use in patients.

As bacteria embedded in the biofilm are up to 1000-fold more resistant to antibiotics compared to planktonic bacteria,^[84]^ use of antibiotics either systemically or in oral rinses and site application are unable to eliminate or manage the biofilm bacteria adequately.^[85,86]^ This has implications both with natural teeth and also periodontal issues developing around dental implants leading to periimplantitis.^[87]^

It has been well documented that cardiac endocarditis or valvular infection can be produced by the bacteria in oral biofilm. Prevention in those patients with current cardiac issues should be managed with premedication with an appropriate antibiotic prior to any dental treatment to prevent seeding of the oral bacteria systemically that can worsen present cardiac issues. Patients with kidney disease, should have their periodontal condition evaluated and treated to eliminate potential oral biofilm contribution to that kidney issue. Those patients also should be educated as those with other systemic health issues as outlined of the connection between their oral health and their general (systemic) issues and addressing those will aid in improving overall health.

## 17. Conclusion

Oral biofilm is recognized as a much more complex material functioning through coordination of bacteria within a protective slime matrix. Extensive data and research have demonstrated that the oral biofilm causing periodontal disease has distant systemic affects and has been connected to numerous medical conditions which is supported in the literature. But the absence of oral bleeding on probing or when brushing does not rule out the presence of a periodontal condition or oral biofilm in the gingival sulcus and a dental evaluation is recommended to aid in eliminating any potential systemic effects from any oral biofilm present.

The health conditions examined here are not the only ones associated with periodontal disease and a full review of all conditions was beyond the scope of this article. Periodontal treatment is evolving to be a major component of full-body medical care and controlling the associated biofilm yields better overall systemic health. Yet frequently there is a lack of coordination between the physician and dentist in management of the oral biofilm and any periodontal disease that can be present. The family physician is in a unique position and is often the first healthcare provider to see the patient and manage their systemic issues. Those patients seen by the primary care physician or medical specialist who are being treated for any of the systemic conditions mentioned should be referred to the patient’s dentist to evaluate and treat any periodontal disease present to improve treatment results of systemic disease. Patient’s that have systemic conditions that are not responding to medical treatment can benefit with dental care to reduce the bacterial in the biofilm and their inflammatory products.

## Author contributions

Gregori M. Kurtzman, DDS – Researched the information and wrote the article

Robert A. Horowitz DDS – Review draft and edit

Richard Johnson, MD – Review draft and edit

Ryan A. Prestiano, MD – Researched literature for references for the text

Benjamin I. Klein – Researched literature for references for the text
